# Optical Analysis of the Internal Void Structure in Polymer Membranes for Gas Separation

**DOI:** 10.3390/membranes10110328

**Published:** 2020-11-05

**Authors:** Chiara Muzzi, Alessio Fuoco, Marcello Monteleone, Elisa Esposito, Johannes C. Jansen, Elena Tocci

**Affiliations:** Institute on Membrane Technology (CNR-ITM), Via P. Bucci, 17/C, 87036 Rende, Italy; c.muzzi@itm.cnr.it (C.M.); a.fuoco@itm.cnr.it (A.F.); m.monteleone@itm.cnr.it (M.M.); e.esposito@itm.cnr.it (E.E.); jc.jansen@itm.cnr.it (J.C.J.)

**Keywords:** molecular dynamics, simulation, free volume element size distribution, non-accessible regions, selectivity, glassy polymers, PIM, PEEK, Hyflon^®^

## Abstract

Global warming by greenhouse gas emissions is one of the main threats of our modern society, and efficient CO_2_ capture processes are needed to solve this problem. Membrane separation processes have been identified among the most promising technologies for CO_2_ capture, and these require the development of highly efficient membrane materials which, in turn, requires detailed understanding of their operation mechanism. In the last decades, molecular modeling studies have become an extremely powerful tool to understand and anticipate the gas transport properties of polymeric membranes. This work presents a study on the correlation of the structural features of different membrane materials, analyzed by means of molecular dynamics simulation, and their gas diffusivity/selectivity. We propose a simplified method to determine the void size distribution via an automatic image recognition tool, along with a consolidated Connolly probe sensing of space, without the need of demanding computational procedures. Based on a picture of the void shape and width, automatic image recognition tests the dimensions of the void elements, reducing them to ellipses. Comparison of the minor axis of the obtained ellipses with the diameters of the gases yields a qualitative estimation of non-accessible paths in the geometrical arrangement of polymeric chains. A second tool, the Connolly probe sensing of space, gives more details on the complexity of voids. The combination of the two proposed tools can be used for a qualitative and rapid screening of material models and for an estimation of the trend in their diffusivity selectivity. The main differences in the structural features of three different classes of polymers are investigated in this work (glassy polymers, superglassy perfluoropolymers and high free volume polymers of intrinsic microporosity), and the results show how the proposed computationally less demanding analysis can be linked with their selectivities.

## 1. Introduction

Efficient gas separations may limit pollution by reducing both gas emission and energy consumption, and many of the relevant industrial separations can be optimized by using membrane-based processes. Membrane-based gas separation is a well-consolidated technique for several applications, such as natural gas treatment, vapor recovery and hydrogen recovery, and its full potentialities are far from being exploited in many other sectors [1,2].

Together with gas separation process engineering, a crucial role is played by the design of new materials with improved properties. Consequently, a good understanding of the correlations between the materials properties and their transport mechanisms is required, along with the realization of innovative functional materials with improved properties. In this context, molecular modeling is a valuable tool for understanding the relationship between the chemical structure and the functional properties of a material. In recent years, molecular simulations have reached an impressive development in membrane science, due to their use both in the research of new materials and in the study of the transport and sorption phenomena [3,4,5,6,7,8,9]. Molecular dynamics (MD) simulations can be also considered as a chemical engineering tool, being that MD is part of the “molecular processes–product–process (3PE)” integrated multiscale approach [10].

The transport properties of a polymeric membrane are usually illustrated in terms of the solution–diffusion mechanism [11], via three parameters: solubility (*S*), diffusivity (*D*) and permeability (*P*). The permeability is the product P=D·S, that summarizes both the thermodynamic and the kinetic contributions. The ratio of the permeabilities of two gases is called permeability selectivity ($\alpha_{P}$), and it can also be expressed in terms of solubility selectivity ($\alpha_{S}$) and diffusivity selectivity ($\alpha_{D}$), via:

(1) $$\alpha_{P}=\frac{P_{i}}{P_{j}}=\alpha_{S}\cdot \alpha_{D}=\frac{S_{i}}{S_{j}}\cdot \frac{D_{i}}{D_{j}}$$

where *i* and *j* indicate two different gas species.

Robeson pointed out the trade-off between permeability and selectivity, defining imaginary upper bounds of gas separation performance [12,13], where high selectivity is associated with low permeability, and vice versa. This is not a fixed limit, but over time, better performing polymers have been introduced, and the upper bound has been revisited [14,15]. The strategy for the improvement of membrane materials usually consists of changing the backbones and side chains of the existing polymers via chemical synthesis or modifying the packing capabilities of polymeric chains. Previous works indicate that the incorporation of rigid and bulky structural elements, which decrease the packing efficiency and chain mobility, can increase gas permeability in the glassy state with minimum selectivity losses [16]. Changes of the morphology and the chemical nature of a membrane material allow tuning of the molecular diffusion and sorption selectivity [17,18]. In a theoretical analysis, Freeman [19] points out a clear correlation between the stiffness of high free volume polymers and superior upper bounds performances. The latter are obtained when there is an enhancement in chain stiffness and/or an increase of the solubility selectivity (in order to increase the selectivity) together with an increase of the fractional free volume through an increase of the interchain distance (for maintaining or increasing permeability) [19]. However, the interchain distance cannot exceed a certain limit because the mechanism of diffusion must always be governed by the thermally stimulated segmental motions of the polymer [19].

The total free volume, and the size and shape of the voids, are of paramount importance for the physical chemistry of polymeric membranes [20]. In molecular simulations, voids have been identified with different methodologies [21,22,23,24,25,26,27,28]. Some simulation methods employ geometric algorithms for describing the molecular system as equivalent hard spheres neglecting the energetic interactions among the particles. They use a Delaunay space tessellation or a space discretization in, for instance, cubic grids [29,30,31]. Other methods used to probe free volume make use of energetic interactions between a probe particle and the atoms of the system to detect the cavities [24,32,33]. These remarkable works illustrated a detailed analysis of volumes and volume’s connectivity.

It is well-known that the size selectivity in membranes originates from the presence of bottlenecks between the individual free volume elements [34,35,36]. Nevertheless, little information is available about the precise correlation at the molecular level between the existence of bottlenecks in the geometrical arrangement of polymeric chains and their effect on the transport properties of a polymeric membrane. To the best of our knowledge, only Park et al. in 2014 used MD simulations for the analysis of bottlenecks and selectivities in OH-containing polyimide (HPI) and thermally rearranged polybenzoxazole (TR-PBO) polymers [37]. In our work, we perform a detailed analysis of spatial organization by assessing the non-accessible paths in three different classes of polymers with high rigidity, having each different amounts of free volume and different selectivities. We investigate polymers of intrinsic microporosity (PIMs), Hyflon^®^ superglassy perfluoropolymers and poly(ether ether ketones) (PEEKs). PIMs [38,39] have a pore size <20 Å and are characterized by the inefficient packing of their rigid and contorted chains that lead to high free volume content and interconnected voids. PIMs were responsible for the first revision of Robeson’s upper bound in 2008 [12]. Hyflon^®^ AD are amorphous perfluorinated copolymers of tetrafluoroethylene and 2,2,4-trifluoro-5-trifluoromethoxy-1,3-dioxole [40] that have high chemical, thermal and aging resistance. Hyflons^®^ are extraordinarily inert to solvents and have high free volume (although less than PIMs) and a high permeability [41]. The PEEKs in this work are unsubstituted cardo poly(aryl ether ketone) with a lactone group sticking out of the backbone (poly(oxa-p-phenylene-3,3-phthalido-p-phenylene-oxa-p-phenylene- oxy-phenylene) (PEEK-WC) [42], and two of its derivatives. They have a low fractional free volume (FFV), excellent mechanical toughness, thermo-oxidative stability, solvent resistance and a high glass transition temperature. PEEK-WC has been employed with success in gas separation, biomedical applications and, in its sulfonated form, in fuel cells [43]. It is soluble in few polar solvents and can be cast into flexible tough films of the neat polymer [42] or as mixed matrix membranes [44].

The aim of this work is to design a fast and simple method to identify with minimum computational effort some of the most important physical characteristics that can be linked to the transport properties of the material, with the ultimate goal to roughly predict the performance of new samples without performing time-consuming data production simulations.

Here, an automatic image recognition tool along with a consolidated Connolly probe sensing of space are used to sample the internal conformations of a number of different polymeric molecular models, leading to a qualitative estimation of non-accessible paths in the geometrical arrangement of polymeric chains. The combination of the proposed methods can be used as qualitative and rapid screening of material model to estimate the trend of their diffusion selectivity.

## 2. Materials and Methods

Eight polymers were analyzed, and their model construction has been reported previously [45,46,47,48,49,50] or is reported in the supporting info. Three models for each material were analyzed (except for Hyflon^®^ AD60x, for which two samples were analyzed). The PIMs studied were the archetypal PIM-1 [39], the fluorinated PIM-2 [45] and the functionalized PIM-NH_2_ [46]; in the group of PEEKs, the unsubstituted PEEK-WC was studied along with the dimethyl PEEK-WC (named DMPEEK) and the tetramethyl PEEK-WC (named TMPEEK) [47,48]. The perfluorinated Hyflons^®^ family was investigated in two of the most experimentally investigated grades, namely Hyflon^®^ AD60x and Hyflon^®^ AD80x [49,50].

**Figure 1:**
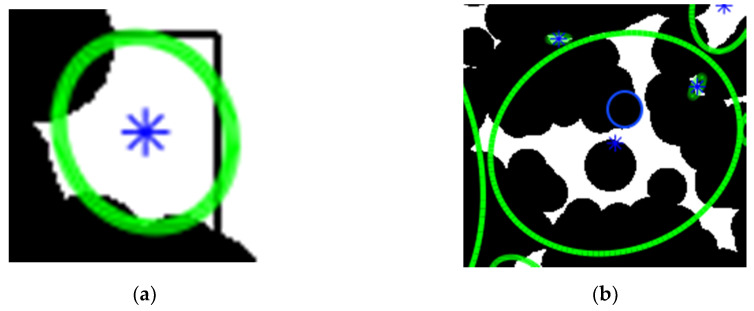
Examples of an ellipse defined on a void by the function “regionprops”: (**a**) a void made of uniformly white pixels, (**b**) a void containing isolated black spots inside, corresponding to fragments of chains. The blue circle highlights a random atom for a visual comparison with the dimensions of space narrowing.

Models were prepared via molecular dynamics dedicated software BIOVIA [51] and opportunely equilibrated to their experimentally determined densities. BIOVIA was used to generate slabs of the boxes, cutting them in the three directions, X, Y and Z. The boxes were sampled in each direction with slabs of a given thickness. A color picture (RGB) of each slab was captured, looking at the wider face of the slabs. Those two-dimensional (2D)-RGB images were then saved as black and white (BW) images. We now had a series of pictures where just three types of information were present: the presence of atoms (black pixels), the absence of atoms (white pixels) and the border of the box (also black pixels). Those 2D-BW images were analyzed via Octave [52], with the “regionprops” function of the “image” package [53]. This function was trained to recognize the edges of objects in high contrast images and, in this method, it was used to trace the border of empty regions made of adjacent white pixels (where black pixels were all around). The empty regions described by those borders were extracted and treated separately, and are generically referred to as voids in the following text. This geometrical analysis was independent from the position of the voids in the box. Therefore, when ellipses overlapped, it had no influence on the way voids were measured. Voids could be both uniformly white regions (Figure 1a) or could incorporate isolated black spots, corresponding to fragments of chains (Figure 1b). In both cases, the voids were isolated and no connectivity analysis could be performed between the different areas contained in a single void [21], nor between different voids. The “regionprops“ function calculated the center of mass of the voids and their internal areas, excluding the holes due to the possible presence of isolated black regions. Then the function traced an ellipse in their centers of mass, with the same area of the voids and with the same normalized second central moment. The code also calculated the eccentricity, and the major and minor axes that would be used in the further analysis. The eccentricity (*ε*) of the ellipse accounts for its elongation and is defined as the ratio of the distance between its foci and its major axis length. The eccentricity value is between 0 (value corresponding to a circle) and 1 (corresponding to a segment). The voids were identified without probes, as in reference [54], and therefore, also very small spaces are monitored. Here, we collected only regions with an area larger than 0.1 Å^2^_,_ smaller than the cross-section occupied by any gas molecule.

All reported data were adequately normalized with respect to the box dimensions in order to be comparable (Table 1).

BIOVIA was also used to evaluate the free volume of the samples at 298 K via the “Atom Volumes & Surfaces” tool [55]. The free volume and its complementary Van der Waals volume (*V_vdW_*), were defined by the Van der Waals surface (*S_vdW_*). *S_vdW_* is the space tangent to the Van der Waals radii of the atoms, when investigated by a zero Connolly radius probe. The Connolly probe is a punctual probe that rolls over the surface of the atoms measuring its dimension through an analytical algorithm [56].

The fractional free volume was calculated according to the Bondi equation [57]:

(2) $$FFV=\frac{V_{tot}-1.3V_{vdW}}{V_{tot}}$$

where *V_tot_* was the volume of the box and 1.3 was a universal packing factor that transformed the Van der Waals volume in the occupied volume (*V_occ_*). The fractional free volume was also calculated from Octave-performed image analysis. In order to go from a two-dimensional area to a three-dimensional volume, each ellipse void of every slab was approximated as an ellipsoid that was traced using the two axes of the ellipses and the thickness of the slabs. The difference $V_{vdW}=V_{tot}-\frac{\sum V_{ellipsoid}}{3}$ was used as Van der Waals volume in the Bondi Equation (2).

## 3. Results and Discussion

### 3.1. Fractional Free Volume Measuments

**Figure 2:**
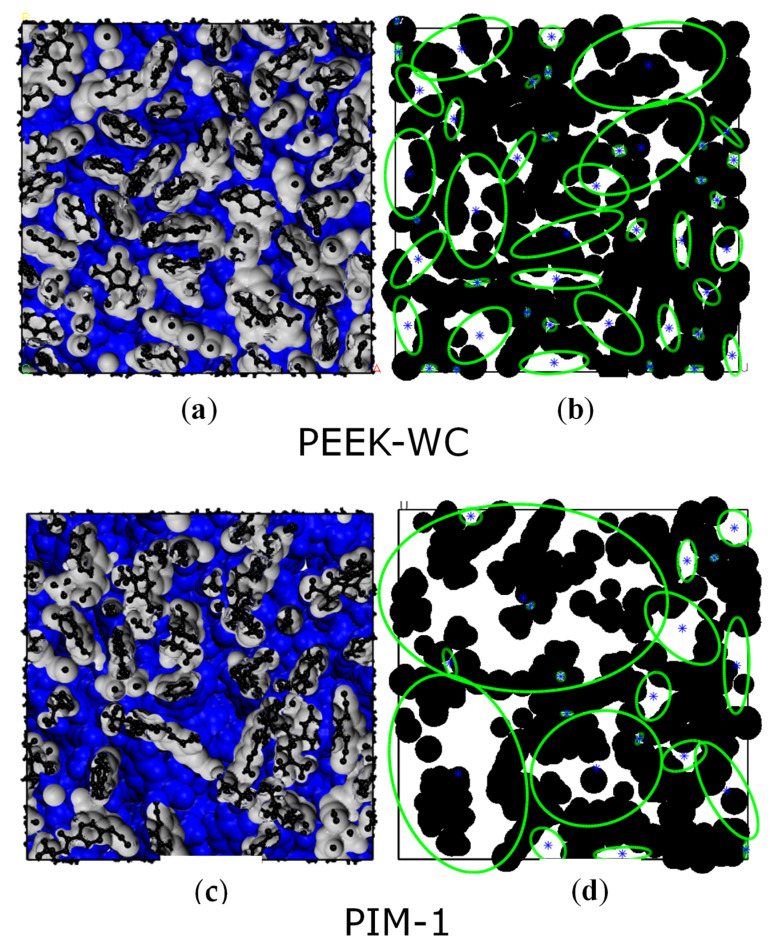
Examples of two types of visualization of the free volume elements: (**a**,**c**) void analysis performed by BIOVIA software via a zero Connolly radius probe [56] rolling over the Van der Waals radii of the atoms in the whole volume of the box; surfaces are colored in grey where facing the atoms and in blue facing the void space; (**b**,**d**) void recognition in a single slab performed via Octave “regionprops” function; atoms are colored in black and displayed with their Van der Waals radii, void space is colored in white and all light and visual effects are disabled to better mark the distinction between filled and void spaces in the boxes. Note: it is not the same surface seen in the left panels, because figures (**a**,**c**) report a lateral snapshot of the analyzed volume as a whole, while figures (**b**,**d**) report the output of the analysis performed on a random two-dimensional (2D) image of a slab extrapolated from the total volume.

Figure 2 reports some pictures of the PEEK-WC polymer with a low fractional free volume (FFV) and of PIM-1 with a high FFV. Figure 2a,c reports some snapshots of the FFV evaluation with a zero Connolly radius probe on the whole volume of the simulation box. Figure 2b,d shows how the automatic image recognition analysis detects voids in the 2D-BW images and reduces them to ellipses. Due to the slab sampling technique that affects depth perception, little information on the actual depth of a void element is obtained in this case. Various thicknesses were tested for slab sampling, and we noticed that 3 Å thick slabs have a depth perception that allows isolating a couple of atomic layers. These 3 Å thick slabs have the right amount of filled space necessary to obtain a sharp perimeter of voids, and we concluded that 3 Å is the best compromise between sharp perimeters and depth sight.

**Figure 3:**
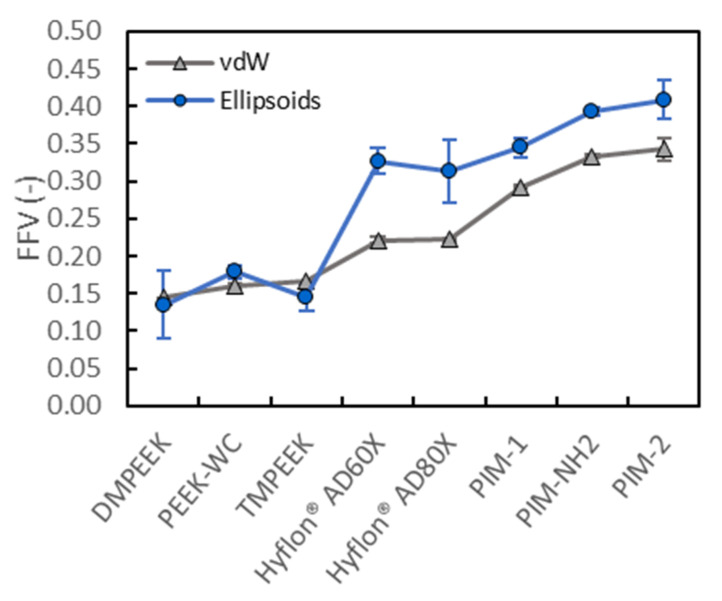
Fractional free volume of all samples calculated with BIOVIA via a zero radius Connolly probe rolling over the surface defined by the vdW radii of the atoms (grey triangles) and via the ellipsoids approximation on the data from image analysis performed using Octave (blue circles). The data points represent the average values of three samples for each material, except for Hyflon^®^ AD60 (two samples). The error bars are always displayed, but sometimes they are smaller than the corresponding marker.

Our Van der Waals FFV values for PIM-1 (0.29), PIM-2 (0.34) and Hyflons^®^ (both 0.22) are in good agreement with the FFV values from the literature, calculated using the group contribution methods (respectively, 0.26 [58], 0.34 [45] and 0.23 [59] for both Hyflons^®^). Figure 3 shows the fractional free volume of all investigated polymers, calculated using Equation (2) with both Van der Waals (i.e., Connolly probe sensing) and ellipsoid methods (i.e., automatic image recognition).

The three classes of polymers show very different fractional free volume, in agreement with the literature [60], with PEEKs having the lowest amount, Hyflons^®^ intermediate values and PIMs the highest amount of FFV due to their rigid contorted polymeric structure that leads to inefficient packing. This is also in agreement with previous computational and experimental studies focused on gas diffusion analysis [36], as reported in Supplementary Materials Table S1. The above distinction is always observed using both the methods herein employed, regardless how the volume is probed. When analyzing high free volume materials, differences between the two methods are visible, i.e., ellipsoid FFVs are higher in Hyflons^®^ (32% and 28% for Hyflon^®^ AD60X and Hyflon^®^ AD80X, respectively) and PIMs (15% PIM-1, 15% PIM-NH2 and 16% PIM-2). However, those values are very similar and slightly lower for PEEKs (−6% DMPEEK, 10% PEEK-WC, −14% TMPEEK). Ellipsoids approximation also marks the differences in the free volume of polymers with a similar backbone but with different substituents, and it can thus be used for qualitative analysis. The smallest and largest voids are, respectively, underestimated and overestimated, due to the investigation strategy, i.e., the slab sectioning and the ellipsoids approximation. This happens because some small voids are more likely to be edge parts of a wider void element than isolated void elements. The ellipsoid FFVs of Hyflons^®^ are higher than the corresponding Van der Waals FFVs. The free volume (*V_f_*) is therefore overestimated by ellipsoids approximation, suggesting that there is a bigger portion of thinner voids that negatively influences their FFV mean value.

### 3.2. Voids Complexity

**Figure 4:**
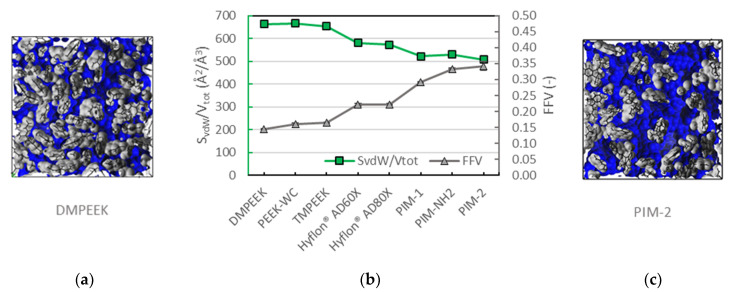
(**a**) Van der Waals surface (*S_vdW_*) of dimethyl poly(ether ether ketones) (DMPEEK); (**b**) the surface area as a fraction on the total volume (green squares) is shown on the primary axis, the fractional free volume (grey triangles) is reported on the secondary axis; (**c**) *S_vdW_* of polymers of intrinsic microporosity (PIM)-2. Surfaces are colored in grey where facing the atoms and in blue facing the void space. Data are averaged on the three investigated samples, except for Hyflon^®^ AD60x (two samples). The error bars are plotted but they are always smaller than the corresponding marker.

Figure 4 compares the Van der Waals surface area, *S_vdW_*, normalized on the total volume (*V_tot_*) of each model, and the associated fractional free volume, both as perceived by the Connolly probe. This comparison provides additional insight into the overall three-dimensional complexity of voids. From the geometrical point of view, it is well-known that a fixed volume can be characterized by a smaller surface as a result of its more regular shape, or a larger surface, indicating an irregular shape (e.g., a complex polyhedron). Directly comparing DMPEEK and PIM-1 reveals that DMPEEK has roughly half of the FFV of PIM-1 with a higher *S_vdW_* than PIM-1. The voids of DMPEEK are therefore smaller than those in PIM-1. This means that DMPEEK contains many small isolated voids or small channels, while there are a series of big and interconnected cavities in PIM-1. No major differences are observed among the three different PEEK samples, and neither between the two Hyflons^®^, even if they have larger voids with more complex shape with respect to the PEEK family. Instead, PIMs show some variations among the different members of the family due to the very different geometrical organization of the chains, which is strictly linked to their chemical structures.

### 3.3. Voids Shape

**Figure 5:**
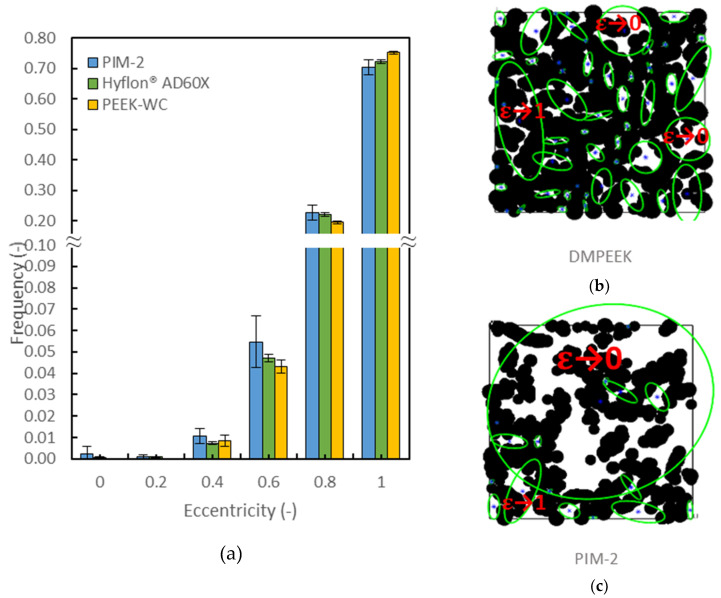
(**a**) Histogram of the eccentricity of the voids in PIM-2, Hyflon^®^ AD60x and poly(ether ether ketones) (PEEK-WC) as the ratio on the total number of voids, averaged on the different boxes (**b**,**c**). Examples of the eccentricities of voids in a slab of DMPEEK and PIM-2.

The overall eccentricity (*ε*) of the ellipses in each 2D slab for the materials is reported in Figure 5a. It displays a probability distribution of the eccentricity of the voids. The sampling technique counts all the enclosed regions of voids once, both if they have small or large dimensions. Almost all the voids of the PEEKs have a small size, while PIMs also contain some big and interconnected voids, in which many void elements are linked into one. Due to the sampling technique, the ε of those big voids (as the biggest one in Figure 5c), goes to 0 and are no more identifiable in the first bin of Figure 5a.

The histogram reports three examples of a general behavior. The eccentricity is almost 1 for all samples, which means that the largest portions of voids are elongated and rod-like, both of small and large dimensions. A smaller portion of voids shows a more circular symmetry (*ϵ*→0), and they are either very small and round (e.g., the smallest voids of DMPEEK in Figure 5b) or wide and spread irregularly over the slab plane (e.g., the biggest void of PIM-2 in Figure 5c).

PEEKs tend to be more elongated than PIMs and Hyflons^®^. The relatively small differences in frequency occur because some small voids of PIMs and Hyflons^®^ are enclosed in a large void element lying in the first bins of the histogram instead of the last.

### 3.4. Void Analysis and Selective Separations

**Figure 6:**
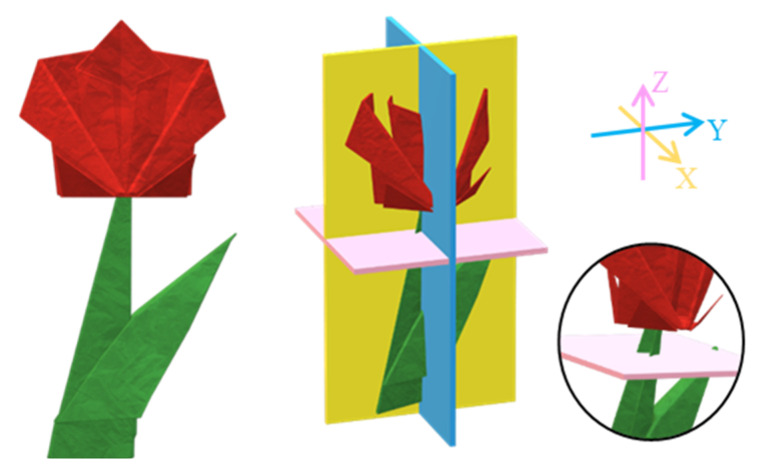
Example of three-dimensional (3D) sampling of a geometrical complex object. The circle highlights how a small section is also perceived. The arrows indicate the normal of the planes.

For irregularly shaped voids with a broad extension in multiple slabs, information about their major and minor dimensions is obtained by multiple sampling of the space in three dimensions. This is schematically displayed with the example in Figure 6. Sampling of the red origami flower in the directions X and Y gives the biggest extension of the shape, whereas sampling in the Z direction provides information on the smallest section of the stem. Therefore, when sampling the free space of a polymeric model, an apparently small void could either be a small isolated void or a bottleneck of a bigger interconnected void.

**Figure 7:**
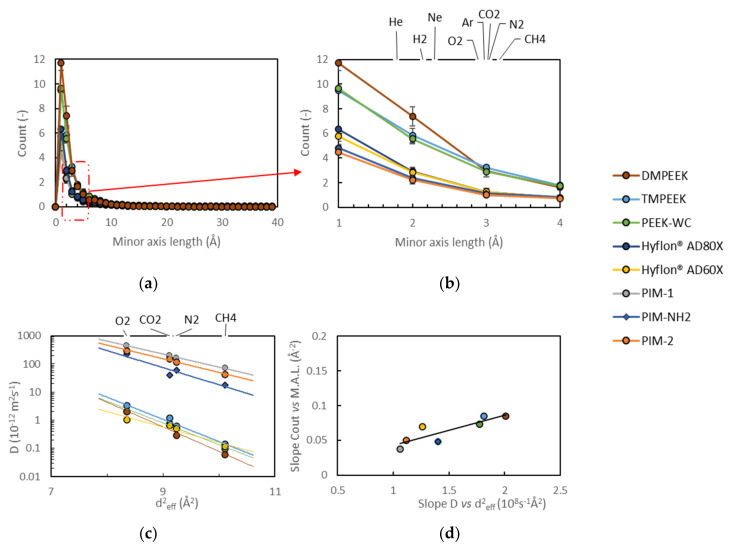
(**a**) The occurrence of minor axis length of all voids counted per 1 nm^3^ volume and averaged on three samples, for each material, except for Hyflon^®^ AD60x and (**b**) a zoom in the range of gas diameters. The error bars are sometimes smaller than marker points. (**c**) Correlation of the diffusion coefficients with the effective gas diameters according to Teplyakov and Meares [61]. (**d**) Correlation of the absolute slopes of the sections between 3 and 4 Å of the lines in Figure 7b and the slope in Figure 7c.

The presence of a bottleneck in the available space of the polymer can prevent a big gas molecule passage as compared to a small gas molecule, introducing the selectivity. The geometrical hindrance to movement is clearly not the only player in determining the gas transport properties of the analyzed samples, but it appears to be a deterministic feature. In this work, all minor axis dimensions of the detected ellipses, whose lengths fall in the range of penetrant gas molecule diameters (Teplyakov and Meares diameters are used [61], reported in Table S1), were counted in order to predict if a gas molecule may pass through the polymer or if it will be blocked. Figure 7a reports the number of the minor axis dimensions of the ellipses (obtained from void recognition) in a volume element of 1 nm^3^, sampled in a histogram of 40 bins. A zoom of the section of Figure 7a corresponding to gas molecule diameters is reported in Figure 7b. The curves allow to estimate how many minor axes of voids with the same dimension of a gas molecule there are in each sample. Those dimensions are large enough for the passage of certain gases but they might block the passage of larger gases. For example, voids with minor axis lengths of the same dimension of neon are accessible also to helium and hydrogen, whilst they are non-accessible to the other bigger gases, thus blocking their passage. The general trend is: the bigger the gas molecule, the less the available space to move across the sample.

In the same class of materials, there is almost the same amount of small void elements, which explains their similar transport properties (at least where non-preferential interaction occurs between the gas molecule and the chain itself). The bigger difference in height in Figure 7b is associated to PEEKs, which are roughly twice as high as PIMs. Hyflons^®^ are once more a middle ground when compared to PEEKs and PIMs. The variation in the number of minor axis lengths is significant compared to the size of the volume element considered and contribute to the differences observed in the transport properties of the materials.

Figure 7c shows the correlation between the squared effective diameter of the penetrant gases and their diffusion coefficients in the investigated polymers. A steeper slope in this figure indicates a higher size selectivity of the polymer [36]. This slope should depend on the voids in the size range of the gas molecules of interest, i.e., with a minor axis length near 3 Å (for O_2_, N_2_, CO_2_ and CH_4_). Indeed, this is illustrated in Figure 7d, by the correlation between the slopes of the curves in Figure 7b in the range from 3 to 4 Å, and the corresponding slopes in Figure 7c, which are a measure of the size selectivity of the polymers. It must be noted that smaller molecules H_2_ and He were not included in this analysis, because they were shown to be subject not only to transport by the solution–diffusion mechanism, but also to Knudsen-like pore flow in high free volume polymers [36].

For screening or prediction of the properties of a new polymer for gas separation membranes, the analysis of Figure 7b alone is sufficient only if a huge difference in terms of free volume are involved. However, the analysis of the complexity made with the observation of the Van der Waals surface area normalized on the total volume of each model strengthens these results and helps to form a preliminary idea of the type of voids that could be gained.

Once this analysis is optimized and automatized, it will quickly highlight some promising candidates when performed on a large number of samples. The method is qualitative but fast: general indications on the occurrences of inaccessible paths and the complexity of the empty spaces can be obtained at the same time. On the other hand, the limitations are the missing details on the distribution and connectivity of the individual voids, obtainable from previously reported methods [21,24,28,29,30,31,32,33]. Our idea is that once the screening highlights the most promising materials, the other more detailed methods previously cited can be used for a complete treatment of the free volume, or in perspective, a new program can be implemented in order to count the actual bottlenecks.

## 4. Conclusions

The comparison among three groups of glassy materials with different amounts of free volume and permeabilities (PIMs, Hyflons^®^ and PEEKs) was successfully performed with the proposed methodology consisting of an automatic image recognition tool along with a consolidated Connolly probe sensing of space. The automatic image recognition analysis draws a picture of the number of constrictions along the path of a gas molecule though the polymers. The number of constrictions with respect to the diameter of gas molecules is strongly correlated with the diffusion selectivity, $\alpha_{D}$. The Connolly probe sensing of space gives additional details on the complexity of voids. As a result, this strategy provides a fast and versatile tool for screening promising materials in silico, by quickly linking their void’s size with their size selectivity. As an independent tool, it offers quick insight into the properties of novel materials without strong computational effort and, in combination with more powerful simulation methods, it allows the first screening of the most interesting materials for further investigation.

## Figures and Tables

**Table 1:** Structures of the investigated polymers with the lateral dimension of the simulation boxes used.

Name	Structure	Simulation Boxes Lateral Dimension (nm)	References
PIM-1	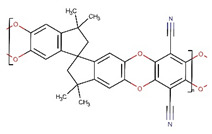	3.999 ± 0.004	[39]
PIM-2	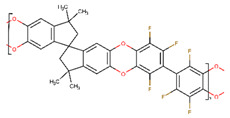	4.33 ± 0.03	[45]
PIM-NH_2_	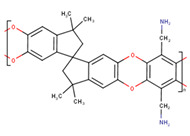	3.9105 ± 0.0045	[46]
PEEK-WC	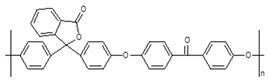	3.90104 ± 0.00002	[47,48]
DMPEEK	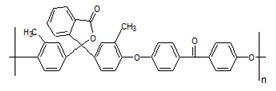	3.97634 ± 0.00001	[48]
TMPEEK	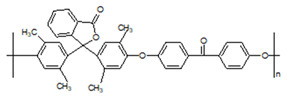	4.103847 ± 0.000005	[48]
Hyflon^®^ AD60x	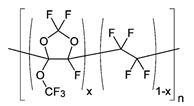 with x = 0.6	4.92 ± 0.04	[49,50]
Hyflon^®^ AD80x	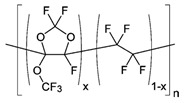 with x = 0.8	4.953 ± 0.004	[49,50]

Hyflon^®^ image is reprinted with permission from [50]. Copyright 2009 American Chemical Society.

## Supplementary Materials

The following are available online at https://www.mdpi.com/2077-0375/10/11/328/s1. Supporting info Table S1: The model preparation of PIM-1.
