# Supplementary material for: Optical Analysis of the Internal Void Structure in Polymer Membranes for Gas Separation
Source: Membranes (Basel). 2020 Nov 5;10(11):328. doi: 10.3390/membranes10110328 (PMC7694385; doi:10.3390/membranes10110328)
Supplement: Supplementary file 1 [file membranes-10-00328-s001.pdf]

membranes

Supplementary Materials

Optical Analysis of the Internal Void Structure in Polymer Membranes for Gas Separation

Chiara Muzzi, Alessio Fuoco, Marcello Monteleone, Elisa Esposito, Johannes C. Jansen and Elena Tocci *

Institute on Membrane Technology (CNR-ITM), Via P. Bucci, 17/C, 87036 Rende (CS), Italy; c.muzzi@itm.cnr.it (C.M.); a.fuoco@itm.cnr.it (A.F.); m.monteleone@itm.cnr.it (M.M.); e.esposito@itm.cnr.it (E.E.); jc.jansen@itm.cnr.it (J.C.J.)

* Correspondence: e.tocci@itm.cnr.it; Tel.: +39-0984-492038

Model preparation of PIM-1

PIM-1 was created with BIOVIA software [1] as a 30-monomers long polymer. The structure was equilibrated via Biovia Forcite's module tool, with COMPASS force-field, force-field assigned charges, Atom bases summation methods, 22.5Å cutoff and 1Å spline. 50 simulation boxes were filled with 3 chains of PIM-1 and 400 Argon atoms at temperature of 298K and a density of 1.5g cm-3 (with a ramp density starting from 0.1 g cm-3), Amorphous Cell tool in BIOVIA was used to perform the calculation. Argon atoms were inserted as spacers to allow a uniform growth of polymeric chains in the boxes and avoid harsh differences in morphology, i.e. very dense and/or partially empty regions within the box. Two boxes were selected and further equilibrated from a preliminary test of their morphology. The initial boxes dimensions were about (43x43x43) Å. Some cycles of Argon atoms deleting and downscaling were performed until all Argon atoms were deleted and density reached about 1.1 g cm-3. The sample was allowed to move through a NVT (with constant number of atoms, constant volume and constant temperature) dynamics for 1ns at 600K and 0.5ns at 400K. Finally, an NPT (with constant number of atoms, constant pressure and constant temperature) dynamics of 5ns at 289K was performed. At this stage the sample did not shown any mayor variation in its density that reached the equilibrium value of 1.082g cm-3 (the experimental density of PIM-1 is 1.069 g cm-3).

Table 1. Experimental diffusivities, solubilities and permeabilities of the investigated materials.

	PIM-1 [2]	PIM-2 [3]	PIM-NH_2 [4]	PEEK-WC	TM-PEEK	DM-PEEK	Hyflon AD60 [5]	Hyflon AD80
				D $[cm^2/s \cdot 10^{-8}]$				
He[1]	7120	5150	5170				2630	
H_2	5760	3870	3970				1500	
O_2	452	297	223	2.15[6]	3.31[6]	1.96[6]	103	
CO_2	199	152	39.3	0.75[6]	1.22[6]	0.64[6]	64.7	82[7]
N_2	165	114	59.8	0.62[6]	0.52[6]	0.30[6]	49.3	
CH_4	70	42.3	17.9	0.10[6]	0.14[6]	0.06[6]	11.6	10.65[7]
				S $[cm^3STP\ /cm^3bar]$				
He	0.19	0.20	0.174				0.10	
H_2	0.61	0.59	0.579				0.09	
O_2	3.54	3.20	3.02	0.33[6]	0.35[6]	0.33[6]	0.51	
CO_2	48	32.5	36.1	2.74[6]	3.36[6]	3.05[6]	1.95	1.5[7]
N_2	3.52	3.03	2.88	0.23[6]	0.27[6]	0.24[6]	0.40	
CH_4	13.7	11.5	12.7	0.64[6]	0.97[6]	0.82[6]	1.32	0.74[7]
				P $[Barrer]$				
He	1830	1400	1200				339	430[7]
H_2	4710	3020	3070	11.7[8]	21.5[8]	10.3[8]	169	210[7]
O_2	2140	1270	895	0.95[8]	1.55[8]	0.87[8]	69.1	67[9]
CO_2	12800	6600	1890	2.73[8]	5.44[8]	2.6[8]	166	150[7]
N_2	773	460	230				26.2	24[7]
CH4	1280	650	303				20.1	12[9]

[1]Atoms are ordered according to their diameters, using T-M [10] values (He 1.78Å, H_2 2.14 Å, O_2 2.89 Å, CO_2 3.02 Å, N_2 3.04 Å and CH_4 3.18 Å).

References

1. *Dassault Systèmes BIOVIA*; Materials Studio; Accelrys Software Inc., 2013;
2. Williams, R.; Burt, Luke.A.; Esposito, E.; Jansen, J.C.; Tocci, E.; Rizzuto, C.; Lanč, M.; Carta, M.; McKeown, Neil.B. A highly rigid and gas selective methanopentacene-based polymer of intrinsic microporosity derived from Tröger's base polymerization. *J. Mater. Chem. A* **2018**, *6*, 5661–5667, doi:10.1039/C8TA00509E.
3. Fuoco, A.; Satilmis, B.; Uyar, T.; Monteleone, M.; Esposito, E.; Muzzi, C.; Tocci, E.; Longo, M.; De Santo, M.P.; Lanč, M.; et al. Comparison of pure and mixed gas permeation of the highly fluorinated polymer of intrinsic microporosity PIM-2 under dry and humid conditions: Experiment and modelling. *J. Membr. Sci.* **2020**, *594*, 117460, doi:10.1016/j.memsci.2019.117460.
4. Mason, C.R.; Maynard-Atem, L.; Heard, K.W.J.; Satilmis, B.; Budd, P.M.; Friess, K.; Lanč, M.; Bernardo, P.; Clarizia, G.; Jansen, J.C. Enhancement of CO 2 Affinity in a Polymer of Intrinsic Microporosity by Amine Modification. *Macromolecules* **2014**, *47*, 1021–1029, doi:10.1021/ma401869p.
5. Macchione, M.; Jansen, J.C.; De Luca, G.; Tocci, E.; Longeri, M.; Drioli, E. Experimental analysis and simulation of the gas transport in dense Hyflon® AD60X membranes: Influence of residual solvent. *Polymer* **2007**, *48*, 2619–2635, doi:10.1016/j.polymer.2007.02.068.
6. Wang, Z.; Chen, T.; Xu, J. Gas Transport Properties of Novel Cardo Poly(aryl ether ketone)s with Pendant Alkyl Groups. *Macromolecules* **2000**, *33*, 5672–5679, doi:10.1021/ma9921807.
7. Yavari, M.; Fang, M.; Nguyen, H.; Merkel, T.C.; Lin, H.; Okamoto, Y. Dioxolane-Based Perfluoropolymers with Superior Membrane Gas Separation Properties. *Macromolecules* **2018**, *51*, 2489–2497, doi:10.1021/acs.macromol.8b00273.
8. Xu, J.; Wang, Z.; Chen, T. Gas Separation Properties of Modified Poly(ether ketone[sulfone]) with Phthalic Side Group. In *Polymer Membranes for Gas and Vapor Separation*; ACS Symposium Series; American Chemical Society, 1999; Vol. 733, pp. 269–276 ISBN 978-0-8412-3605-9.

9. Arcella, V.; Colaianna, P.; Maccone, P.; Sanguineti, A.; Gordano, A.; Clarizia, G.; Drioli, E. A study on a perfluoropolymer purification and its application to membrane formation. *J. Membr. Sci.* **1999**, *163*, 203–209, doi:10.1016/S0376-7388(99)00184-2.
10. Teplyakov, V.; Meares, P. Correlation aspects of the selective gas permeabilities of polymeric materials and membranes. *Gas Sep. Purif.* **1990**, *4*, 66–74, doi:10.1016/0950-4214(90)80030-O.
